# The Prevalence of Polypharmacy and Potentially Inappropriate Medications and Its Relationship with Cognitive Status in Portuguese Institutionalized Older Adults: A Cross-Sectional Study

**DOI:** 10.3390/ijerph19052637

**Published:** 2022-02-24

**Authors:** Catarina Caçador, Edite Teixeira-Lemos, Jorge Oliveira, João Pinheiro, Luís Teixeira-Lemos, Fernando Ramos

**Affiliations:** 1 Faculty of Pharmacy, University of Coimbra, Azinhaga de Santa Comba, 3000-548 Coimbra, Portugal; cacasabel@hotmail.com; 2 ESAV, Polytechnic Institute of Viseu, 3500-606 Viseu, Portugal; etlemos3@gmail.com (E.T.-L.); joliveira@esav.ipv.pt (J.O.); 3 CERNAS-IPV Research Centre, Polytechnic Institute of Viseu, 3504-510 Viseu, Portugal; 4 Faculty of Medicine, University of Coimbra, 3000-548 Coimbra, Portugal; jpinheiro@fmed.uc.pt; 5 Nuclear Medicine Department, Centro Hospitalar e Universitário de Coimbra (CHUC), 3004-561 Coimbra, Portugal; 11652@chuc.min-saude.pt; 6 REQUIMTE/LAQV, R. D. Manuel II, Apartado, 55142 Oporto, Portugal

**Keywords:** nursing homes, polypharmacy, potentially inappropriate medications, cognitive assessment

## Abstract

The aim of this study was to evaluate the prevalence of polypharmacy and potentially inappropriate medications (PIMs) in a population of older adults living in nursing homes. Furthermore, we also intended to assess the possible association between polypharmacy, potentially inappropriate medications and cognitive impairment in institutionalized older adults. A cross-sectional study analyzed data from 193 nursing home residents in the district of Viseu, Portugal, between September 2018 and June 2019, with a mean age of 82.4 ± 6.2 years (ranging from 65 to 95 years old); 72.5% (*n* = 140) were female participants. Major polypharmacy was presented in 80.8% of the study population, who took 7.6 ± 3.3 drugs per day. Using the Beers Criteria, we found that 79.3% took PIMs. There was a positive association between polypharmacy and PIM (*p* < 0.001), showing that higher medicines intake increased the number of PIMs. Polypharmacy was not associated with the functionality of the older adults to perform activities of daily living, but was associated with cognitive impairment. The older adults with lower scores on the Mini Mental State Examination (MMSE) took more drugs (*p* = 0.039) and used more PIM (*p* < 0.001). Moreover, patients taking five or more prescription drugs per day (major polypharmacy) consuming any psychiatric, gastrointestinal or oral antidiabetic agents (regardless of whether they were considered potentially inappropriate or not) had higher odds of displaying cognitive impairment than those who did not (*p* < 0.05). Older adult residents of the studied nursing homes were potentially affected by polypharmacy and inappropriate polypharmacy. This observation reveals the need to adopt and implement strategies that make drug therapy more adequate and safer for older adults.

## 1. Introduction

The aging process combined with the increase in life expectancy leads to higher prevalence of multimorbidity worldwide [1]. Portugal, with the third highest aging index in Europe in 2018, is no exception [2]. Currently and worldwide, 81.5% of people over 85 years of age experience multimorbidity (defined by two or more chronic diseases) [3], which consequently increases drug consumption and the risk of polypharmacy [4]. Polypharmacy is defined as the use of multiple medications by a patient; there is no standardized minimum threshold for the number of medicines one can take in a day [5]. Age is a risk factor for polypharmacy with 20 to 30% of older adults taking more than four medications. The process of institutionalization worsens the situation and, on average, nursing home residents take more than eight drugs per day [6].

Polypharmacy, besides increasing health care costs, is also associated with other negative consequences [7]. Individuals who take multiple medications are at higher risk of adverse drug events, drug interaction and potentially inappropriate medication (PIM) [7]. Older people are even more susceptible to these effects due to age-related physiological changes that can alter drug pharmacokinetics and pharmacodynamics, impacting hepatic elimination and renal excretion [8]. It is estimated that 51% of the reported adverse drug events were presumable preventable [9]. Furthermore, in older people, polypharmacy is also often associated with physical dysfunction and cognitive decline [10].

A fine line separates polypharmacy and the risk of PIM in the older adults. To meet several clinical guidelines, polypharmacy is required [6]. For instance, three medications are often required to manage symptoms of heart failure or control blood pressure, and at least two medications are required for efficient glucose control [11]. On the other hand, as PIM is defined as drugs that are ineffective or with a poor benefit/risk ratio, evidence showed that a reduction in drug ingestion decreased the risk of PIM without compromising health status [12,13].

To minimize the occurrence of PIM and, subsequently, inappropriate polypharmacy, it is essential to consider the risk–benefit ratio of each drug. The Beers Criteria is one of the most used methods to assess PIM use [8]. These guidelines are frequently revised by the American Geriatric Society, listing the medicines that should be typically avoided by older adults in ordinary conditions or under specific situations, such as when suffering from certain diseases or conditions [14]. The updated version of the Beers Criteria includes a separate PIM list for people with dementia and delirium, recognizing the importance of management of these medications in this older adult subset. It has also been reported that patients with dementia are prescribed an average of 5 to 10 drugs, with most treatments indicated for other comorbid medical conditions [15]. For instance, the use of multiple medications in this population, particularly anticholinergic and sedative agents, may worsen memory loss and increase functional impairment [16].

Yet, even with the application of diverse tools, studies have demonstrated that PIM is still a concern among older people and that this specific population is the most frequently exposed to PIM [17,18]. Estimations indicate that this practice ranges from 18% to 48.7% in outpatients, 13% to 54% in hospitalized patients and 37% to 67% in nursing home residents [19]. The implementation of simple and effective action projects for drug therapy management is essential to avoid and control PIM prescription, and thereby improve older adults’ life quality [20,21]. The study performed by Simões et al. (2019) concerning the prevalence of potentially inappropriate medication in the older adult population attending primary care in Portugal provided the first approach to the situation [22]. Nevertheless, data on Portuguese institutionalized older adults are lacking. Therefore, our study aimed to examine the prevalence of polypharmacy and potentially inappropriate medications in a population of older adults living in nursing homes of the city of Viseu, in central Portugal. Furthermore, we also intended to assess the possible association between polypharmacy, potentially inappropriate medications and cognitive impairment in our population of institutionalized older adults.

## 2. Material and Methods

### 2.1. Patient and Public Involvement

Patients and the public were not involved in the design, conduct, reporting, or dissemination plans of our research.

### 2.2. Subject Recruitment and Data Collection

The present work is a cross-sectional study performed with data collected from 14 long-term care institutions for older adults/nursing homes that agreed to collaborate in the study and are located in the city of Viseu, Portugal or within a distance of 20 km (12 mi) from the city. These nursing homes represent 90% of the total nursing homes in the city and they could be either private or supported by the government. To be included in the study, these nursing homes should have more than 25 beds and be supported by health professionals (a nurse and a general practitioner).

**Figure 1:**
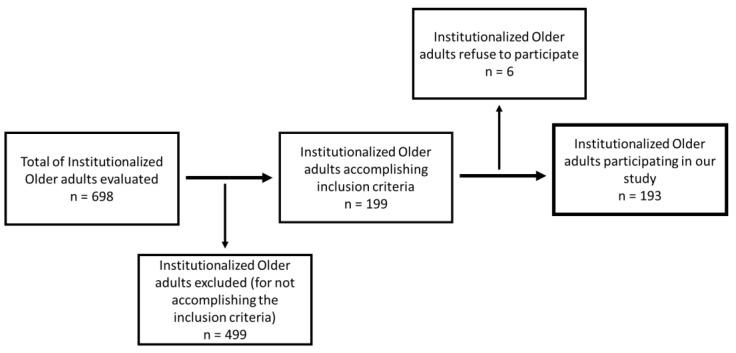
Participant inclusion flowchart.

A total of 698 patients of all the nursing homes who had agreed to enroll in the study were then contacted and informed about the study. Inclusion criteria were: (1) age ≥ 65 years old, (2) residence in nursing homes at least for the past 12 weeks prior to the study, (3) Barthel Index (BI) ≥ over or equal to 40 points, (4) ability to walk (with or without technical devices), (5) understand written and spoken Portuguese and (6) acceptance to participate in the study. The exclusion criteria were: (1) temporary residence in the institution or residence for <3 months prior to the study, (2) exhibit cognitive and behavioral deterioration suggesting inability to understand or give informed consent or had a diagnosis of Alzheimer’s disease and (3) decline to participate in the survey. Only 193 participants met the eligibility criteria. However, six declined to participate. Figure 1 provides a participant inclusion flowchart.

The included institutions are residential structures for the elderly (ERPI) that have their own kitchen, so all have a cook and kitchen assistants. In addition to management positions, they also have social workers and operational assistants/home helpers who provide support to all the elderly. These institutions also have the support of social workers, physical education teachers and a health team (nurse, physiotherapist and a doctor).

The study was approved by the Ethics Committee of the Polytechnic Institute of Viseu (Ref. 01/sub/2021) and conforms to the provisions of the Declaration of Helsinki (as revised in Brazil 2013). All participants gave their written informed consent to participate in the study. The reporting of this study conforms to the Strengthening the Reporting of Observational Studies in Epidemiology (STROBE) statement [23].

Following written informed consent, a trained researcher collected the data and performed the anthropometric measures. Data were collected from September 2018 to June 2019. Data collection included demographic and socioeconomic characteristics (sex, age, educational level, marital status), functional status for instrumental activities of daily living (ADL), cognitive function and number and type of medication.

The formal education demographic was categorized according to the number of school attendance years: illiterate (0 years), 1–11 years, >11 years. The information collected was recorded in a computerized database designed for this purpose. This database was anonymized prior to any analyses to ensure data protection.

### 2.3. Body Mass Index (BMI)

Anthropometric measurements, such as weight and height, were taken in order to assess nutritional status. Height (rounded to the nearest 0.1 cm) was measured using a measuring tape, with the participants standing upright against a wall without shoes. Participants were weighed with a digital chair scale to the nearest 0.1 kg. An adequate nutritional status is fundamental for the proper management of polypharmacy and BMI is a gold-standard indicator of malnutrition. Therefore, BMI was calculated as weight (kg)/(height (m))^2^, and classified according to the method of Lipschitz et al. [24].

### 2.4. Performance in Activities of Daily Living (ADL)

The Barthel Index (BI) is used to evaluate the functional ability of the older adults in 10 ADL (ambulation, chair/bed transfers, bathing self, personal hygiene, stairs climbing, feeding, toilet use, bowel control, bladder control, dressing) [25]. The index is calculated by summing the response value to each of these items. It has been validated in the Portuguese population [26]. The higher the score following Barthel Index assessment, the greater the likelihood for the patient to be able to live at home, independently, with varying degrees of help and care. The BI total score ranges from 0 to 100 points and classifies the individual’s level of dependence as follows: score under 20, totally dependent; score between 20 and 39, very dependent; 40 and 59, partially dependent; 60 and 79, minimally dependent; and 80 and 100, able to live independently [26].

### 2.5. Cognitive Performance

Cognitive status was evaluated by the Mini Mental State Evaluation (MMSE), validated for the Portuguese population [27]. This is one of the most widely used instruments for cognitive impairment screening. It includes 30 items and assesses temporal and spatial orientation, working memory, recall, attention, arithmetic capacity, and linguistic and visual motor skills. The maximum score is 30 points (one point per correct item). Portuguese cut-offs for cognitive impairment were applied, according to the number of years the participant attended school: illiterate ≤15; 1–11 years of study ≤ 22 points; and >11 years of study ≤ 27 points [27].

### 2.6. Polypharmacy and PIMs

Information regarding medication was collected based on patient records provided by the nursing staff. The medicines used by the participants were classified into pharmacologic groups based on those defined on the Portuguese *“Therapeutic record”.* Polypharmacy was divided into minor (2 to 4 daily medicines) and major (5 or more daily medicines). Considering that five or more medications should be taken regularly for a longer period of time, time-limited medications such as antibiotics were excluded from the calculation of total number of medications taken by each patient. Furthermore, supplements and vitamins that do not need a prescription (e.g., calcium, multivitamin) were also excluded. However, supplements that require a prescription such as vitamin B12 and potassium chloride were included.

For each participant, PIMs were assessed based on the Beers Criteria [8], independently of patient diagnosis or conditions once individual’s clinical history was not provided.

Authors used the last updated (2019) version of the criteria released by the American Geriatric Society for Potentially Inappropriate Medication Use in Older Adults [14].

This tool has already undergone several revisions, the last being in 2019, and includes six tables: listing “potentially inappropriate medications in older patients apart from the clinical condition” Table 2, “medication use in older adults due to drug–disease or drug–syndrome interactions that may exacerbate the disease or syndrome” Table 3, “potentially inappropriate medications in older patients considering the clinical condition” Table 4, “potentially inappropriate medications—drugs to be used with caution in older adults” Table 5, “potentially clinically important drug–drug interactions that should be avoided in older adults” Table 6, and “medications that should be avoided or have their dosage reduced with varying levels of kidney function in older adults” listed in Table 7. The criteria were applied using only the information contained in the sociodemographic characteristics (age and gender) and patients’ current medication list (i.e., international non-proprietary names, dosages, pharmaceutical forms, and regime of each medicine). The information of 2019 Beers Criteria Table 6 was ignored because creatinine clearance data were not available in the patients’ medical records. Classification was performed by two independent authors (C.C and E.T.L.). When discrepancies existed, a decision was achieved by consensus meetings between the authors.

### 2.7. Statistical Analysis

Analysis of data involved descriptive statistics such as mean, standard deviation and frequencies of sociodemographic variables (continuous and categorical) such as age, gender, educational level and marital status, and BMI, BI and MMSE classes. The gender effects were analyzed by Chi square tests according to the above variables and polypharmacy and potentially inappropriate medications variables. The association between presence or absence of polypharmacy and nutritional status, functionality for daily living, cognition and the presence of potentially inappropriate medication was achieved with Chi square tests. Binary logistic regression analysis was used to understand the influence of the factors age, gender, polypharmacy, the presence of PIMs and pharmacological classes on cognitive impairment (MMSE scores). The collected data were analyzed using IBM SPSS Statistics software, version 26.0. The level of significance of *p* ≤ 0.05 was considered for all statistical analyses.

## 3. Results

### 3.1. Characteristics of the Participants

A total of 193 nursing home residents were recruited, with a mean age of 82.4 ± 6.2 years (ranging from 65 to 95 years old); 72.5% (*n* = 140) were female participants. The participants presented low educational level (illiterate or 11 years of schooling = 88.0%) and most of them were widowed, separated or divorced (73.6%). BMI values ranged from 16.0 kg/m^2^ to 43.3 kg/m^2^, with a mean value of 28.5 ± 5.0 kg/m^2^. Roughly 6.7% of the subjects were classified as underweight (BMI < 22 kg/m^2^), while 58.5% were considered overweight (BMI > 27 kg/m^2^). There were no significant differences by gender in the three BMI categories. For ADL, most of the population (74.6%) were able to live independently, according to their BI scores. A total of 70 participants (36.3%) presented some degree of cognitive impairment according to the MMSE screening, with women having a worse cognitive performance than men (*p* = 0.015). Sociodemographic data as well as BMI, BI and MMSE scores are summarized in Table 1.

### 3.2. Polypharmacy and PIM

Participants were found to take an average (±standard deviation) of 7.6 ± 3.3 drugs daily. Polypharmacy was identified in 97.9% of the older adults. Major polypharmacy (five or more medications per day) was present in 80.8% of the participants. Of the 193 older adults, a total of 153 (79.3%) were prescribed at least one PIM independent of diagnoses or condition (Table 2).

**Figure 2:**
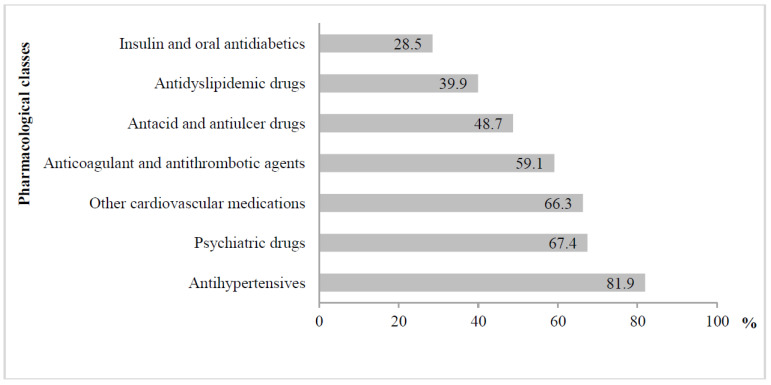
Drug classes most frequently used by the participants of the study (%).

The most commonly prescribed medications were antihypertensives, followed by psychiatric agents (67.4%) and other cardiovascular medications (66.3%) (Figure 2). Figure 2 also shows the consumption of other pharmacological subsets in lower percentages.

**Figure 3:**
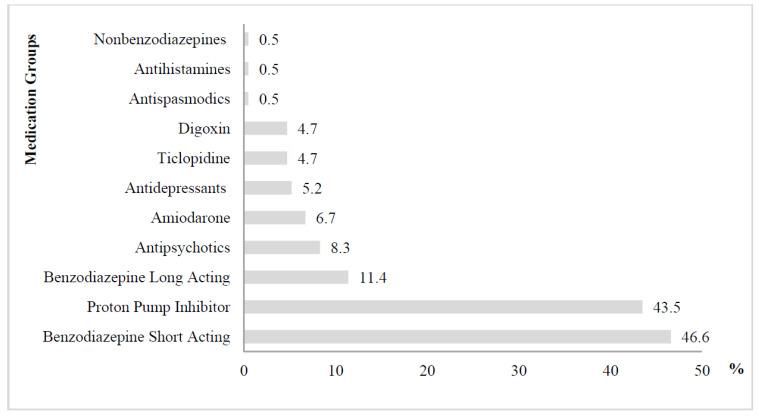
Therapeutic classes and drugs to be avoided for most older adults according to the Beers Criteria *(independent of diagnoses or conditions*).

According to the Beers Criteria, the pharmacological groups of most found PIMs were short-acting benzodiazepines (alprazolam) (46.6%) and proton-pump inhibitors (omeprazole) (43.5%) (Figure 3). Moreover, long-acting benzodiazepines (diazepam) and antipsychotics such as quetiapine, loxapine, and haloperidol (in descending order) were found.

Table 3 presents information on the magnitude of associations between polypharmacy and the different health domains analyzed as well as the presence of PIMs. Our analysis revealed a significant association between major polypharmacy and cognitive impairment (*p* = 0.039). However, functionality for basic daily living activities (BI) does not seem to be affected by polypharmacy in this group. Major polypharmacy and PIM were significantly associated (*p* < 0.001) (Table 3).

To understand the possible association between cognitive impairment (as measured by MMSE scores) and several factors such as age, gender, polypharmacy and the use of some drug classes, binary logistic regression analysis was performed (Table 4). In our population, female participants had a higher likelihood of having cognitive impairment than their male counterparts (*p* = 0.017). Similarly, patients taking five or more prescription drugs per day (major polypharmacy) or consuming any psychiatric, gastrointestinal or oral antidiabetic agents (regardless of whether they were considered potentially inappropriate or not) had higher odds of displaying cognitive impairment than those who did not.

## 4. Discussion

Polypharmacy and PIM are a concern all over the world [28,29]. Institutionalization and cognitive decline seem to be the predictive risk factors for these conditions [30,31]. It is estimated that approximately 60–70% of long-term nursing home residents have some degree of cognitive impairment [32,33], and that, within this population, more than 20% take five or more medications [34,35]. It is noteworthy that a high prevalence of PIM has been reported among patients with dementia, including Alzheimer’s disease [36]. To the best of our knowledge, this is the first study performed in nursing homes of the city of Viseu, in central Portugal, reporting the prevalence of polypharmacy and PIM and their possible association with cognitive impairment in a population of older adults.

Our study identified major polypharmacy (five or more drugs) in 80.8% of the studied nursing home older adults, 79.3% of whom used at least one PIM. The number of consumed drugs per day is different among nursing home residents across countries. Different physicians’ attitudes when prescribing treatments for complex patients may explain such differences [37,38]. On average, the number of daily medications described in this study accorded with the SHELTER project data obtained across 57 institutions from eight different European countries (7.0 ± 3.6, average (SD)) [33]. Our results pointed out a significant risk factor for receiving inappropriate drugs when major polypharmacy occurs, especially in women. Our study adopted the most updated Beers Criteria, which allow the identification of drug inappropriateness in the older adult population. The Beers Criteria are a practical tool for screening potential drug-related problems and to guide drug prescription in different health care settings. Short-acting benzodiazepines were the principal PIM consumed by the participants, followed by proton-pump inhibitors. Previous studies had highlighted the same inappropriate exposure to benzodiazepines in institutionalized older adults, with rates reaching 30% [17,39]. Benzodiazepines seem to be widely used by Portuguese older adults. Nevertheless, their use is often inappropriate and calls for concern. They are used for the treatment of insomnia and anxiety, and their consumption is often related with decreased cognitive impairment [40,41] and the high rate of falls and hip fracture [42].

The present study included institutions considered as residential structures for the elderly (ERPI), welcoming from completely autonomous elderly people to elderly people with various types of physical dependence. Here, older adults benefit from the intervention of multidisciplinary technical teams. All have a nursing team integrated into the technical staff of the institution and also have the support of a general practitioner who visits the institutions regularly. The design of these structures may partially justify the elevated presence of independent older adults in ADL (74.6%) with no impaired cognition (63.7%).

Importantly, cognitive impairment was associated with an increased rate of major polypharmacy. A survey across community-dwelling older adults in Japan also showed a similar association, where polypharmacy was present in 48.3% of individuals with deficits in cognition [43]. Moreover, as well as in our investigation, polypharmacy was associated with decreased MMSE scores and an increased risk of cognitive impairment in other studies [35,36,44]. In fact, by increasing the number of prescribed medicines, the risk of unexpected events is increased, and cognitive function may be impacted [7].

Additionally, our results showed that elders who took at least one PIM were more likely to have lower MMSE scores. This fact is extremely important as some PIM may adversely affect cognition. Such cases should be closely followed up in order to understand whether their cognitive deficits are a consequence of neurodegenerative processes or were caused (or worsened) by any PIM [45,46,47]. On the other hand, the intake of these drugs helps to control neuropsychiatric symptoms, delirium episodes, aggressive behavior, and agitation, conferring protection to both the patient and the caregiver. Thus, whether categorized as PIM or not, our older adults with cognitive decline were more likely to use psychotropic medicines, oral antidiabetics, antacid and antiulcer medicines. Longitudinal studies should be further implemented to assess the impact of PIM in the cognitive function of older adults. Additionally, a more detailed stratification of MMSE scores and inclusion of participants with psychiatric diseases and dementia will lead to demonstration of a more accurate relationship among these variables. In fact, determining the causality/effect of cognitive function versus PIM will be essential to scrutinize whether cognitive function is affected by using PIMs, especially medications that affect cognition, or if patients with cognitive impairment and other comorbidities have the real need of more medication use and more PIM.

This study highlights the importance of the involvement of a pharmacist as part of the multidisciplinary health care team in nursing homes. Pharmacists are in a unique position to close monitoring of the number of medicines prescribed to older adults, as well as their suitability. Several systematic reviews have examined the effects of pharmacist-led interventions in nursing homes and have demonstrated promising results [48,49]. Considering the above results, embedding a pharmacist as part of the multidisciplinary health care team to conduct medication management activities alongside nurses, careers and doctors would be important to improve the wellbeing of older adults in nursing homes [50].

Our study had some limitations. First, the small sample size. Then, the cross-sectional design of our study, besides not allowing extrapolation to big populations, only collects the data at a single moment in time. In this way, it was not possible to assess PIMs when they are only considered inappropriate when used for more than a specific period. The Beers Criteria can only identify potentially inappropriate medications, not actually inappropriate medications. In addition, in the design of the study, the inclusion criteria were established to minimize the impact on the elder. Highly dependent individuals in which anthropometric measures are difficult to perform were excluded as well as those with cognitive impairment in which external interventions may disturb and condition their normal routines. Thus, these implemented inclusion criteria might reduce some correlation strength. Nevertheless, this study characterized multiple indexes of older adults (BI for ADLs, MMSE for cognitive impairment and BMI for nutritional status), allowing a complete and integrated vision of the studied population.

## 5. Conclusions

Our investigation expands the knowledge on polypharmacy and the use of PIMs and their attendant risk for the cognitive status of institutionalized older adults in Portugal, where data in this field are scarce. This study found a population with reduced prevalence of cognitive and functional disability but with a very high incidence of polypharmacy and PIM. We believe that this finding will serve to alert health professionals (doctors, pharmacists, nurses) of the need to regularly and methodically control the prescriptions of older adults. In addition, non-pharmacological interventions should be considered in order to improve or maintain functionality and cognition in institutionalized older adults. We believe that this finding will serve to alert health professionals (doctors, pharmacists, nurses) of the need to regularly and methodically control the prescriptions of older adults. The prevalence of polypharmacy and PIM may increase the likelihood of cognitive impairment. Thus, minimizing and preventing these situations perhaps may be beneficial to the cognitive function of older adults. Reducing polypharmacy in addition to disease control may delay the harmful effects on cognition induced by age and, therefore, this study also reinforces the value of constant verification of both polypharmacy and PIM in older adults with cognitive impairment. Overall, this study aims to shed light and increase awareness of the importance of polymedication and PIM among older adults, with inherent implications for policy makers.

## Figures and Tables

**Table 1:** Main baseline characteristics of the study population by gender (N = 193).

	Total Number (%)	Female Number (%)	Male Number (%)	*p*-Value
Educational Status				
Illiterate	68 (35.2)	52 (76.5)	16 (23.5)	0.350
1–11 years	102 (52.9)	74 (72.5)	28 (27.5)	
>11 years	23 (11.9)	14 (60.9)	9 (39.1)	
Marital Status				
Single	23 (11.9)	19 (82.6)	4 (17.4)	0.003 *
Married	28 (14.5)	13 (46.4)	15 (53.6)	
W/S/D	142 (73.6)	108 (76.1)	34 (23.9)	
BMI				
Underweight	13 (6.8)	8 (61.5)	5 (38.5)	0.654
Normal	67 (34.7)	49 (73.1)	18 (26.9)	
Overweight	113 (58.5)	83 (73.5)	30 (26.5)	
BI				
Partially dependent (score 40–59)	13 (6.7)	11 (84.6)	2 (15.4)	0.246
Minimally dependent (score 60–79)	36 (18.7)	29 (80.6)	7 (19.4)	
Independent (80–100)	144 (74.6)	100 (69.4)	44 (30.6)	
MMSE				
Cognitive impairment	70 (36.3)	58 (82.9)	12 (17.1)	0.015 *
Without cognitive impairment	123 (63.7)	82 (66.7)	41 (33.3)	

Note: W/S/D—widow/separated/divorced; BMI—body mass index; BI—Barthel Index; MMSE—Mini Mental State Examination. Illiterate individuals with MMSE score ≤15, individuals with 1 to 11 years of study and MMSE score ≤22 and individuals with more than 11 years of study and MMSE score ≤27 were considered to have cognitive impairment. Percentages in brackets. Statistical comparisons between genders, according to Chi square. * Statistically significant differences (*p* < 0.05).

**Table 2:** Polypharmacy and potentially inappropriate medications (PIMs) by gender.

	Total	Female	Male	*p*-Value
Polypharmacy				
Minor Polypharmacy (2–4 drugs)	37 (19.2)	26 (70.3)	11 (29.7)	0.731
Major Polypharmacy (≥5 drugs)	156 (80.8)	114 (73.1)	42 (26.9)	
Potentially Inappropriate Medications				
0 drugs	40 (20.7)	25 (62.5)	15 (37.5)	0.046 *
1 drug	75 (38.9)	50 (66.7)	25 (33.3)	
2 drugs	52 (26.9)	44 (84.6)	8 (15.4)	
≥ 3 drugs	26 (13.5)	21 (80.8)	5 (19.2)	

Percentages in brackets. Statistical comparisons between genders, according to Chi square. * Statistically significant differences (*p* < 0.05).

**Table 3:** Association between polypharmacy and functionality for daily living, cognition and the presence of potentially inappropriate medication.

	Presence of Major Polypharmacy Number (%)		
	No	Yes	*p*-Value
Age			
65–76	11 (26.8)	30 (73.2)	0.430
77–86	18 (18.0)	82 (82.0)	
87–99	9 (17.3)	43 (82.7)	
Gender			
Female	26 (18.6)	114 (81.4)	0.526
Male	12 (22.6)	41 (77.4)	
BI			
Partially dependent (40–60)	1 (7.7)	12 (92.3)	
Minimally dependent (60–79)	4 (11.1)	32 (88.9)	0.149
Independent (80–100)	33 (22.9)	112 (77.1)	
MMSE			
Cognitive impairment	8 (11.4)	62 (88.6)	0.029 *
Without cognitive impairment	30 (24.4)	93 (75.6)	
PIM			
No	24 (60.0)	16 (40.0)	<0.001 *
Yes	14 (9.2)	139 (90.8)	

BMI—body mass index; BI—Barthel Index; MMSE—Mini Mental State Examination; PIM—potentially inappropriate medication. Illiterate individuals with MMSE score ≤ 15, individuals with 1 to 11 years of study and MMSE score ≤22 and individuals with more than 11 years of study and MMSE score ≤27 were considered to have cognitive impairment. *N* = number of patients. Percentages in brackets. Statistical comparisons between older adults with major polypharmacy (≥4 drugs) or without polypharmacy by Chi square. * Statistically significant differences (*p* < 0.05).

**Table 4:** Binary logistic regression analysis of potential association between cognitive impairment (MMSE scores) and the factors age, gender, major polypharmacy, PIM and various pharmacological classes in institutionalized older adults.

	OR	CI95%	*p*-Value
Age	1.000	0.954–1.049	1.000
Male	0.414	0.200–0.855	0.017 *
Female	2.417	1.169–4.994	0.017 *
Major polypharmacy	2.391	1.026–5.571	0.043 *
Presence of PIM	0.823	0.440–1.537	0.541
Antihypertensives	1.300	0.594–2.845	0.511
Psychiatric medication	2.347	1.193–4.614	0.013 *
Other cardiovascular medication	0.958	0.516–1.781	0.893
Anticoagulant and Antithrombotic Agents	1.062	0.584–1.932	0.842
Antacid and Antiulcer Drugs	1.867	1.030–3.384	0.040 *
Antidyslipidemic Agents	0.567	0.306–1.050	0.071
Oral Antidiabetics	2.060	1.017–4.173	0.045 *

OR—odds ratio; CI—confidence interval. * Statistically significant correlations (*p* < 0.05).

## Data Availability

The datasets used and/or analyzed during the current study are available from the corresponding author on reasonable request.

## References

[1] Atella V., Piano Mortari A., Kopinska J., Belotti F., Lapi F., Cricelli C., Fontana L. (2019). Trends in age-related disease burden and healthcare utilization. Aging Cell.

[2] Fundação Francisco Manuel dos Santos (2018). PORDATA—Ageing Index. https://www.pordata.pt/en/Europe/Ageing+index-1609.

[3] Lavan A.H., Gallagher P.F., O’Mahony D. (2016). Methods to reduce prescribing errors in elderly patients with multimorbidity. Clin. Interv. Aging.

[4] Villén N., Guisado-Clavero M., Fernández-Bertolín S., Troncoso-Mariño A., Foguet-Boreu Q., Amado E., Pons-Vigués M., Roso-Llorach A., Violán C. (2020). Multimorbidity patterns, polypharmacy and their association with liver and kidney abnormalities in people over 65 years of age: A longitudinal study. BMC Geriatr..

[5] Masnoon N., Shakib S., Kalisch-Ellett L., Caughey G.E. (2017). What is polypharmacy? A systematic review of definitions. BMC Geriatr..

[6] Levy H.B., Barney K.F. (2016). Pharmacology, pharmacy, and the aging adult: Implications for occupational therapy. Occupational Therapy with Aging Adults: Promoting Quality of Life through Collaborative Practice.

[7] Maher R.L., Hanlon J., Hajjar E.R. (2014). Clinical consequences of polypharmacy in elderly. Expert Opin. Drug Saf..

[8] Moreira F.S.M., Jerez-Roig J., Ferreira L.M.B.M., Dantas A.P.Q.M. (2020). Uso de medicamentos potencialmente inapropriados em idosos institucionalizados: Prevalência e fatores associados. Cien. Saude Colet.

[9] Gurwitz J.H., Field T.S., Avorn J., McCormick D., Jain S., Eckler M., Benser M., Edmondson A.C., Bates D.W. (2000). Incidence and preventability of adverse drug events in nursing homes. Am. J. Med..

[10] Gnjidic D., Hilmer S., Blyth F.M., Naganathan V., Waite L., Seibel M., McLachlan A., Cumming R., Handelsman D.J., Le Couteur D. (2012). Polypharmacy cutoff and outcomes: Five or more medicines were used to identify community-dwelling older men at risk of different adverse outcomes. J. Clin. Epidemiol..

[11] Cadogan C.A., Ryan C., Hughes C.M. (2016). Appropriate Polypharmacy and Medicine Safety: When Many is not Too Many. Drug Saf..

[12] Linsky A., Simon S.R., Stolzmann K., Meterko M. (2018). Patient attitudes and experiences that predict medication discontinuation in the Veterans Health Administration. J. Am. Pharm. Assoc..

[13] Zechmann S., Trueb C., Valeri F., Streit S., Senn O., Neuner-Jehle S. (2019). Barriers and enablers for deprescribing among older, multimorbid patients with polypharmacy: An explorative study from Switzerland. BMC Fam. Pract..

[14] American Geriatrics Society (2019). American Geriatrics Society 2019 Updated AGS Beers Criteria^®^ for Potentially Inappropriate Medication Use in Older Adults. J. Am. Geriatr. Soc..

[15] Redston M.R., Hilmer S.N., McLachlan A.J., Clough A.J., Gnjidic D. (2018). Prevalence of Potentially Inappropriate Medication Use in Older Inpatients with and without Cognitive Impairment: A Systematic Review. J. Alzheimer’s Dis..

[16] Parsons C. (2017). Polypharmacy and inappropriate medication use in patients with dementia: An underresearched problem. Ther. Adv. Drug Saf..

[17] Primejdie D.P., Bojita M.T., Popa A. (2016). Potentially inappropriate medications in elderly ambulatory and institutionalized patients: An observational study. BMC Pharmacol. Toxicol..

[18] Nothelle S.K., Sharma R., Oakes A.H., Jackson M., Segal J.B. (2017). Determinants of Potentially Inappropriate Medication Use in Long-Term and Acute Care Settings: A Systematic Review. J. Am. Med Dir. Assoc..

[19] Clyne B., Bradley M.C., Hughes C., Fahey T., Lapane K.L. (2012). Electronic Prescribing and Other Forms of Technology to Reduce Inappropriate Medication Use and Polypharmacy in Older People: A Review of Current Evidence. Clin. Geriatr. Med..

[20] Mekdad S.S., Alsayed A.A. (2019). Quality improvement project to reduce drug-related problems (DRPs) and potentially inappropriate medications (PIMs) in Geriatrics Cardiac Clinic in Saudi Arabia. Can. Geriatr. J..

[21] Lenander C., Bondesson A., Viberg N., Beckman A., Midlöv P. (2018). Effects of medication reviews on use of potentially inappropriate medications in elderly patients; A cross-sectional study in Swedish primary care. BMC Health Serv. Res..

[22] Simões P.A., Santiago L.M., Maurício K., Simões J.A. (2019). Prevalence Of Potentially Inappropriate Medication In The Older Adult Population Within Primary Care In Portugal: A Nationwide Cross-Sectional Study. Patient Prefer. Adherence.

[23] Von Elm E., Altman D.G., Egger M., Pocock S.J., Gøtzsche P.C., Vandenbroucke J.P. (2008). The Strengthening the Reporting of Observational Studies in Epidemiology (STROBE) statement: Guidelines for reporting observational studies. J. Clin. Epidemiol..

[24] Lipschitz D.A. (1994). Screening for nutritional status in the elderly. Prim. Care.

[25] Sinoff G., Ore L. (1997). The Barthel activities of daily living index: Self-reporting versus actual performance in the old-old (> or =75 years). J. Am. Geriatr. Soc..

[26] Araújo F., Ribeiro J., Oliveira A., Pinto C. (2007). Validação do Índice de Barthel numa amostra de idosos não institucionalizados. Revista Portuguesa Saúde Pública.

[27] Guerreiro MP S.A., Silva A.P., Botelho M.A., Leitão O., Castro-Caldas A., Garcia C. (1994). Adaptação à População Portuguesa da Tradução do Mini Mental State Examination (MMSE). Rev. Port. Neurol..

[28] Santana I., Duro D., Lemos R., Costa V., Tábuas-Pereira M., Simoes M., Freitas S. (2016). Mini-mental state examination: Avaliação dos novos dados normativos no rastreio e diagnóstico do défice cognitivo. Acta Med. Port..

[29] Khezrian M., McNeil C.J., Murray A.D., Myint P.K. (2020). An overview of prevalence, determinants and health outcomes of polypharmacy. Ther. Adv. Drug Saf..

[30] Payne R.A. (2016). The epidemiology of polypharmacy. Clin. Med. (Lond.).

[31] O’Dwyer M., Peklar J., McCallion P., McCarron M., Henman M. (2016). Factors associated with polypharmacy and excessive polypharmacy in older people with intellectual disability differ from the general population: A cross-sectional observational nationwide study. BMJ Open.

[32] Jokanovic N., Tan E.C.K., Dooley M.J., Kirkpatrick C.M., Bell J.S. (2015). Prevalence and Factors Associated With Polypharmacy in Long-Term Care Facilities: A Systematic Review. J. Am. Med. Dir. Assoc..

[33] Onder G., Liperoti R., Fialova D., Topinkova E., Tosato M., Danese P., Gallo P.F., Carpenter I., Finne-Soveri H., Gindin J. (2012). Polypharmacy in nursing home in Europe: Results from the SHELTER study. J. Gerontol. A Biol. Sci. Med. Sci..

[34] Seitz D., Purandare N., Conn D. (2010). Prevalence of psychiatric disorders among older adults in long-term care homes: A systematic review. Int. Psychogeriatr..

[35] Jyrkkä J., Enlund H., Lavikainen P., Sulkava R., Hartikainen S. (2011). Association of polypharmacy with nutritional status, functional ability and cognitive capacity over a three-year period in an elderly population. Pharmacoepidemiol. Drug Saf..

[36] Rawle M.J., Cooper R., Kuh D., Richards M. (2018). Associations Between Polypharmacy and Cognitive and Physical Capability: A British Birth Cohort Study. J. Am. Geriatr. Soc..

[37] Delgado J., Bowman K., Clare L. (2020). Potentially inappropriate prescribing in dementia: A state-of-the-art review since 2007. BMJ Open.

[38] Birke H., Jacobsen R., Jønsson A.B., Guassora A.D.K., Walther M., Saxild T., Laursen J.T., Vall-Lamora M.H.D., Frølich A. (2020). A complex intervention for multimorbidity in primary care: A feasibility study. J. Comorb..

[39] Fried T.R., Tinetti M.E., Iannone L. (2011). Primary care clinicians’ experiences with treatment decision making for older persons with multiple conditions. Arch. Intern. Med..

[40] De Oliveira Martins S., Soares M.A., Foppe Van Mil J.W., Cabrita J. (2006). Inappropriate drug use by Portuguese elderly outpatients—Effect of the Beers criteria update. Pharm. World Sci..

[41] Siddiqui T.G., Cheng S., Gossop M., Kristoffersen E.S., Grambaite R., Lundqvist C. (2020). Association between prescribed central nervous system depressant drugs, comorbidity and cognition among hospitalised older patients: A cross-sectional study. BMJ Open.

[42] Weston A.L., Weinstein A.M., Barton C., Yaffe K. (2010). Potentially inappropriate medication use in older adults with mild cognitive impairment. J. Gerontol. A Biol. Sci. Med. Sci..

[43] Aspinall S.L., Springer S.P., Zhao X., Cunningham F.E., Thorpe C.T., Semla T.P., Shorr R.I., Hanlon J.T. (2019). Central Nervous System Medication Burden and Risk of Recurrent Serious Falls and Hip Fractures in Veterans Affairs Nursing Home Residents. J. Am. Geriatr. Soc..

[44] Niikawa H., Okamura T., Ito K., Ura C., Miyamae F., Sakuma N., Ijuin M., Inagaki H., Sugiyama M., Awata S. (2017). Association between polypharmacy and cognitive impairment in an elderly Japanese population residing in an urban community. Geriatr. Gerontol. Int..

[45] Oyarzun-Gonzalez X., Taylor K.C., Myers S.R., Muldoon S.B., Baumgartner R.N. (2015). Cognitive decline and polypharmacy in an elderly population. J. Am. Geriatr. Soc..

[46] Loggia G., Attoh-Mensah E., Pothier K., Morello R., Lescure P., Bocca M.-L., Marcelli C., Chavoix C. (2020). Psychotropic polypharmacy in adults 55 years or older: A risk for impaired global cognition, executive function, and mobility. Front. Pharmacol..

[47] Helvik A.-S., Benth J.Š., Wu B., Engedal K., Selbæk G. (2017). Persistent use of psychotropic drugs in nursing home residents in Norway. BMC Geriatr..

[48] Wright D.J., Maskrey V., Blyth A., Norris N., Alldred D.P., Bond C.M., Desborough J., Hughes C.M., Holland R.C. (2020). Systematic review and narrative synthesis of pharmacist provided medicines optimisation services in care homes for older people to inform the development of a generic training or accreditation process. Int. J. Pharm. Pract..

[49] Lee S.W.H., Mak V.S.L., Tang Y.W. (2019). Pharmacist services in nursing homes: A systematic review and meta-analysis. Br. J. Clin. Pharmacol..

[50] Silva C., Ramalho C., Luz I., Monteiro J., Fresco P. (2015). Drug-related problems in institutionalized, polymedicated elderly patients: Opportunities for pharmacist intervention. Int. J. Clin. Pharm..

